# Delayed Presentation of Traumatic Right-Sided Diaphragmatic Hernia after Abdominoplasty

**DOI:** 10.1155/2014/949531

**Published:** 2014-05-08

**Authors:** Caroline C. Jadlowiec, Lois U. Sakorafas

**Affiliations:** ^1^University of Connecticut Integrated General Surgery Residency Program, Farmington, CT 06030, USA; ^2^Department of Surgery, Hartford Hospital, Trauma and Critical Care, Hartford, CT 06102, USA

## Abstract

Traumatic diaphragmatic hernias are rare and challenging to diagnose. Following trauma, diagnosis may occur immediately or in a delayed fashion. It is believed that left traumatic diaphragmatic hernias are more common as a result of the protective right-sided anatomic lie of the liver. If unrecognized, traumatic diaphragmatic injuries are subject to enlarge over time as a result of the normal pressure changes observed between the thoracic and abdominal cavities. Additionally, abrupt changes to the pressure gradients, such as those which occur with positive pressure ventilation or surgical manipulation of the abdominal wall, can act as a nidus for making an asymptomatic hernia symptomatic. We report our experience with a delayed traumatic right-sided diaphragmatic hernia presenting with large bowel incarceration two months after abdominoplasty. In our review of the literature, we were unable to find any reports of delayed presentation of a traumatic right-sided diaphragmatic hernia occurring acutely following abdominoplasty.

## 1. Introduction

Traumatic diaphragmatic hernias are rare and challenging to diagnose. Review of trauma literature finds the overall incidence to be approaching 5 percent with higher percentages observed in penetrating trauma as compared to blunt [1, 2]. Following trauma, diagnosis may occur immediately or in a delayed fashion. Delayed diagnosis is not surprising as diaphragmatic defects remain asymptomatic until there is herniation of abdominal contents into the chest. As per literature report, left traumatic diaphragmatic hernias appear to be more common as compared to the right [3]. This tendency is believed to occur as a result of the protective right-sided anatomic lie of the liver which provides coverage and reduces forceful impact to the right diaphragmatic leaf. We report our experience with a delayed traumatic right-sided diaphragmatic hernia presenting with large bowel incarceration about two months after abdominoplasty.

## 2. Case Presentation

**Figure 1:**
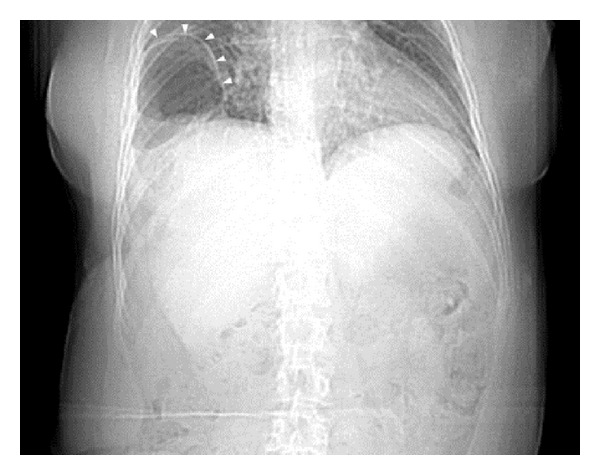
Computer tomography imaging revealing a right-sided diaphragmatic hernia (white arrows).

**Figure 2:**
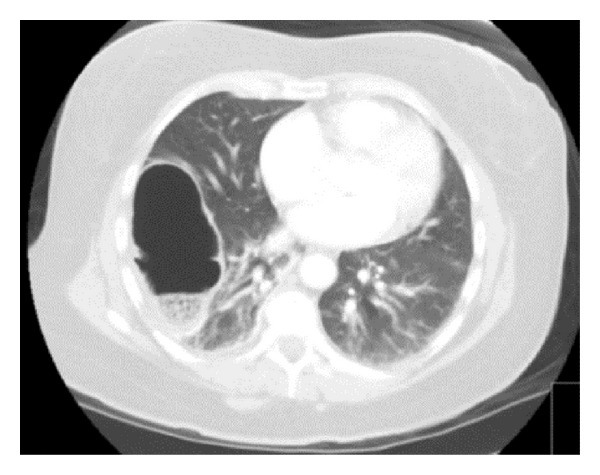
Computer tomography showing protrusion of ascending colon into the right hemithorax.

**Figure 3:**
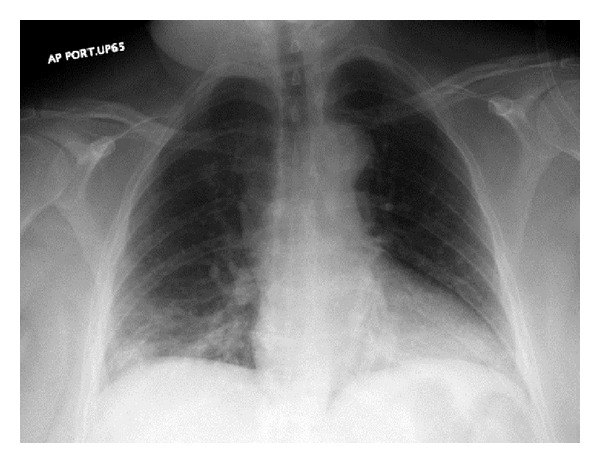
Normal chest radiograph performed prior to abdominoplasty showing no evidence of a diaphragmatic hernia.

**Figure 4:**
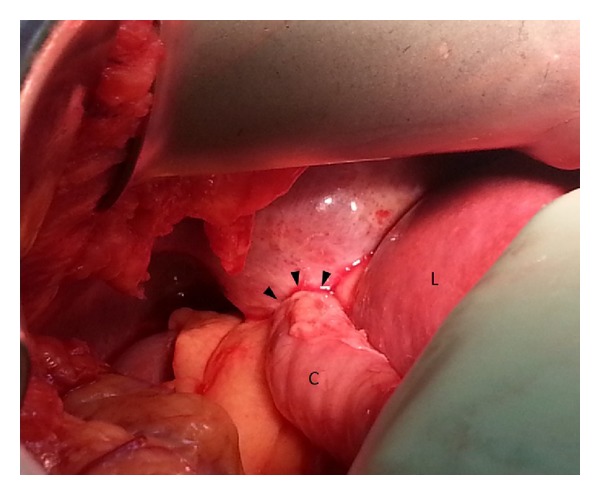
Intraoperative photo showing incarcerated but viable colon (C) next to adjacent liver (L) within a diaphragmatic defect (black arrows).

**Figure 5:**
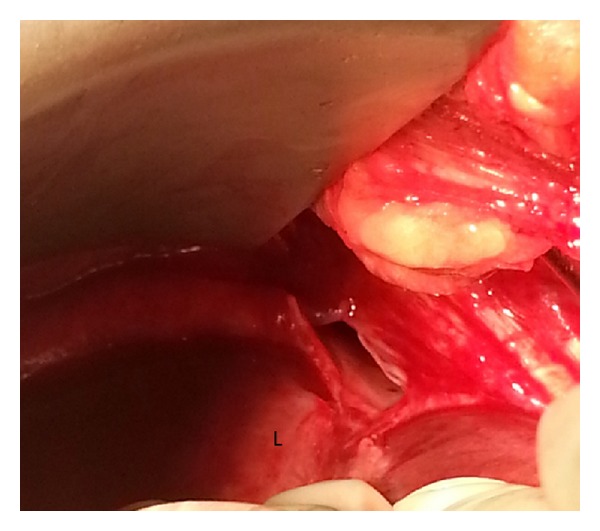
Intraoperative photo showing a 6 cm right-sided diaphragmatic defect; Liver (L).

A 50-year-old female with a recent history of an elective cosmetic panniculectomy and abdominoplasty from 8 weeks earlier presented with acute onset abdominal and right shoulder pain. The patient's past medical history was insignificant other than a remote history of blunt trauma secondary to a motor vehicle collision twenty years earlier. Her vital signs were within normal limits and physical exam revealed right upper quadrant tenderness to palpation. Laboratory results were unremarkable. Abdominal computer tomography imaging revealed a right-sided diaphragmatic hernia (Figure 1) with protrusion of ascending colon into the right hemithorax (Figure 2). Previous chest radiographs performed prior to the abdominoplasty were unremarkable (Figure 3). The patient underwent an emergent laparotomy. Findings in the operating room confirmed incarcerated but viable colon (Figure 4) within a 6 cm diaphragmatic defect (Figure 5). The surgical treatment performed included reduction of hernia contents, transabdominal pleural drainage, and primary repair of the diaphragmatic defect with nonabsorbable suture. The reduced incarcerated transverse colon was hyperemic but viable. Following surgery, the patient had an uneventful hospital course with discharge home on postoperative day four.

## 3. Discussion

Diaphragmatic hernias which present in adulthood are commonly trauma-related; however, other etiologies can also be observed with hiatal and paraesophageal being the most common (Table 1) [4–8]. Traumatic diaphragmatic hernias occur from injury to the musculotendinous membrane and are believed to predominantly occur on the left as a result of the anatomically protective location of the liver. The diaphragm itself is a thin musculoaponeurotic barrier that separates the thoracic and abdominal cavities. Diaphragmatic thickness can be estimated to be between 0.2 mm and 0.9 mm, which makes diagnosis of injury difficult when obvious herniation is not present [9]. Because of the diaphragm's dynamic role in respiration, it is continuously subjected to radial forces. Under normal conditions, a pressure gradient exists between the more negative thoracic cavity and the positive pressure of the abdominal compartment. Over time, secondary to these continuous pressure changes, small unrecognized defects can enlarge, particularly when subjected to increased intraabdominal pressure. In such circumstances, delayed presentation can be observed and has been reported to occur at intervals ranging from weeks to years [1]. Abrupt changes to the pressure gradients have also been noted to result in asymptomatic hernias becoming symptomatic [10]. The observation of an increased incidence of inguinal hernias following abdominoplasty has been reported in the plastic surgery literature [11]. In reviewing our patient's delayed presentation, we accordingly hypothesize that her recent history of an abdominoplasty may have resulted in higher intraabdominal pressures, thus causing her asymptomatic hernia to enlarge and lead to bowel incarceration. Indeed, the true incidence of diaphragmatic hernias is likely underreported secondary to the relatively high incidence of missed diagnoses [12]. This observation appears to be particularly true with regard to right-sided diaphragmatic hernias, thus raising the question if indeed left-sided hernias are more common or rather more easily diagnosed because of symptomatology.

Within the setting of a delayed presentation, diagnosis of a traumatic versus congenital diaphragmatic hernia lies in obtaining a remote history of significant trauma and in assessing the hernia's anatomic location [1]. In completing a thorough medical and surgical history, our patient had indeed sustained a significant lateral impact trauma after being involved in a motor vehicle collision 20 years prior. Anatomically, the majority of traumatic hernias affect the posterolateral diaphragm. This location is similar to that of the pediatric Bochdalek hernia, which likewise occurs in this location. Characteristically, however, Bochdalek hernias present early in the neonatal period with symptoms of respiratory distress. Case reports of adult Bochdalek hernias have been reported but appear to be exceptionally rare [13]. For adults, Morgagni diaphragmatic hernias, positioned anteriorly on the diaphragm, are more common and can be differentiated secondary to location. Operative repair of chronic diaphragmatic hernias is classically approached through the ipsilateral chest. Surgical treatment may be performed either through an open laparotomy or thoracotomy or through laparoscopy or thoracoscopy [2, 14]. A thoracic approach is recommended secondary to concerns over viscera-pleural adhesions and the risk of intrathoracic visceral perforation [14]. In the presence of intestinal obstruction, an abdominal approach may also be required to completely assess viability of organs. We elected to use an abdominal approach because of our patient's acute presentation and the supporting clinical and radiographic findings, which were strongly suggestive of incarcerated bowel with possible ischemia. Review of a chest radiograph performed two months prior to the abdominoplasty was unremarkable (Figure 3). Indeed, intraoperatively, adhesions were not observed and there was no evidence of chronic incarceration. These findings argue in favor of the patient's recent abdominoplasty provoking bowel incarceration into a chronic unrecognized right-sided diaphragmatic defect. Although the type of closure used for diaphragmatic hernias is still a matter of debate, it is generally accepted that most defects can be closed primarily with nonabsorbable suture [15]. Closure with mesh typically is reserved for situations where the defect is too large to be closed primarily. Concerns regarding the routine use of mesh include the potential for mesh erosion into the stomach or esophagus, esophageal stricture formation, mesh migration, and infection [16, 17]. Most surgeons have abandoned the use of nonabsorbable mesh at the esophageal hiatus for these reasons [15]. More recently, biologic mesh has been introduced as an alternative to help buttress the diaphragmatic repairs. Although the use of biologic mesh does not carry the same risk of complications as observed with the tradition polypropylene mesh, newer studies suggest it does not alter long-term hernia recurrence and should accordingly be used at each surgeons' discretion [18].

In summary, despite significant advances in diagnostic technology, traumatic diaphragmatic hernias remain challenging entities. Left-sided hernias are generally believed to be more common, yet we know that the overall incidence of right traumatic diaphragmatic hernias is likely underestimated [2, 14]. Factors resulting in increased intraabdominal pressure can, in fact, reveal missed diaphragmatic defects. We accordingly hypothesize that right-sided diaphragmatic hernias occur at a higher rate than is reported in the literature as the anatomic lie of the liver impedes visceral incarceration and allows them to remain asymptomatic. Factors predisposing to increased intraabdominal pressure may be the impetus required to uncover these missed defects and should be entertained when one encounters a delayed presentation of a traumatic diaphragmatic hernia.

## Figures and Tables

**Table 1:** 

Etiology of Diaphragmatic Hernias
Non-Traumatic
Congenital
Bockdalek
Morgagni
Acquired
Hiatal
Paraesophageal
Iatrogenic
Inflammatory necrosis
Traumatic
Blunt
Penetrating
